# Analysis of Early Post-Radiation Surgical Management of Metastatic Spinal Tumors

**DOI:** 10.3390/jcm14031032

**Published:** 2025-02-06

**Authors:** Sang Yun Seok, Jae Hwan Cho, Hyung Rae Lee, Jae Woo Park, Jin Hoon Park, Dong-Ho Lee, Chang Ju Hwang, Sehan Park

**Affiliations:** 1 Department of Orthopedic Surgery, Daejeon Eulji Medical Center, Daejeon 35233, Republic of Korea; oper251@hanmail.net; 2 Department of Orthopedic Surgery, Asan Medical Center, University of Ulsan College of Medicine, Seoul 05505, Republic of Korea; osdlee@gmail.com (D.-H.L.); basky47@gmail.com (C.J.H.); birdone86@gmail.com (S.P.); 3 Department of Orthopedic Surgery, Korea University Anam Hospital, Seoul 02841, Republic of Korea; drhrleeos@gmail.com; 4 Department of Orthopedic Surgery, Gangneung Asan Hospital, Gangneung 25440, Republic of Korea; yunc0926@gmail.com; 5 Department of Neurosurgery, Asan Medical Center, Seoul 05505, Republic of Korea; jhpark@amc.seoul.kr

**Keywords:** Bilsky grade, metastatic spinal tumor, preoperative radiotherapy, treatment

## Abstract

**Background/Objectives:** Radiotherapy is one of the various treatment options for patients with metastatic spinal tumors (MST). However, it is difficult to say that this is definitely an optimal treatment for MST, and there are several patients who need surgical treatment because pain or neurologic deficits occur even after radiotherapy. Therefore, this study aimed to analyze which patients received early operative treatment after radiotherapy. **Methods:** We included 81 patients who underwent decompression and fusion surgery after radiotherapy for MST. Patients who underwent surgery within 6 months after radiotherapy were classified as the early operation group (group E, *n* = 47), while surgery cases after 6 months after radiotherapy were assigned to the late operation group (group L, *n* = 34). Risk factor analysis using multivariate regression analysis for early operative treatment after radiotherapy was performed. Also, we analyzed the period from radiotherapy to surgery according to the Bilsky grade. **Results:** In multivariate analysis, pathologic fractures and semirigid (thoracic) lesions were more frequent in group E than group L (adjusted odds ratio, 4.282, 10.524; *p* = 0.001, 0.039). In subgroup analysis, there was a difference in the period from radiotherapy to surgery in Bilsky grades above 2 than Bilsky grade 1 (grade 1, 13.6 ± 11.4 months, grade 2, 6.9 ± 6.8 months, grade 3, 6.6 ± 7.5 months; grade 1 vs. 2, *p* = 0.049, grade 1 vs. 3, *p* = 0.047). **Conclusions:** Although the information in this study may only be limited to patients who underwent surgery, early operative treatment after radiotherapy is highly likely for patients with MST accompanied by a Bilsky grade above 2, pathologic fracture and thoracic lesion. In these patients, surgical treatment could be considered as the primary treatment.

## 1. Introduction

Metastatic spinal tumor (MST) can cause severe pain and neurological symptoms in cancer patients [1,2]. As medical treatment advances, the survival rate of cancer patients has increased, and accordingly, the incidence of MST is also increasing [3,4]. MST occurs in about 70% of cancer patients, and only 10–20% of them cause symptoms, causing pain, neurological deficit, and quality of life deterioration [5]. In general, when MST is diagnosed, a multidisciplinary approach of chemotherapy, radiotherapy, and surgery is performed, but recently, innovation in imaging and radiation strategies, including spatially fractionated radiation therapy and radiosurgery, high-performance particle therapy and the use of radio-enhancers, have expanded the indication for radiation treatment, hence delaying the need for surgical debulking [6,7,8,9]. Primary surgery is performed for instability and evolving neurological deficits due to epidural spinal cord compression, whereas radiation treatment as primary strategy is usually prescribed to patients with a low Bilsky score and without instability who have metastatic spine-induced pain as their only primary presenting symptom [1,10].

However, this radiation treatment always does not bring success to all patients, and despite radiation treatment, there are some patients who lead to surgical treatment due to the worsening of neurological symptoms, mechanical pain by the progression of cord compression or further collapse at the lesion of MST. In addition, unlike previous treatments for MST patients, recently surgical treatment has been actively performed for the patient’s life expectancy and quality of life for the rest of their life [11,12]. Therefore, the purpose of this study is to identify the characteristics of patients who underwent surgery at an early time after radiotherapy and to find out in which patients it is advantageous to consider surgery rather than radiotherapy as primary treatment.

## 2. Materials and Methods

### 2.1. Study Design and Patients

This study was retrospectively conducted between March 2014 and January 2020, targeting patients who underwent preoperative radiotherapy among those who underwent surgical treatment for MST. This study was approved by the institutional review board of the authors’ affiliated institutions, which waived the requirement for informed consent because of the retrospective nature of the data analysis (approval no. A20202988). The decision for treatment was based on a consensus in a multidisciplinary center consisting of medical oncologists, radiation oncologists and spine surgeons who took into consideration factors such as life expectancy, performance, symptoms and future systemic treatment plans. In most cases, operative treatment was performed after this consensus, but in some cases, operative treatment was required immediately due to the rapid progression of neurological deficits.

Operations were performed by three spine surgeons (J.H.C., J.W.P. and J.H.P.). As for the surgical method, posterior decompression and fusion of the lesion causing the neurologic deficit were performed. In most cases, two levels above and below were performed, but in patients with metastasis at the one level of cord compression and the adjacent level, three levels above and below were performed to maintain stability. In patients with good general condition and minimal bleeding, an additional corpectomy was performed according to the posterior approach. Indications for surgery are usually uncontrolled mechanical pain and neurological deficits, like motor weakness in the extremities. The exclusion criteria were as follows: (1) other surgery such as separation surgery, vertebroplasty, and kyphoplasty; (2) insufficient chart records of the patients’ treatment; (3) when the stereotactic radiosurgery was performed; (4) if the surgery was performed during radiotherapy; (5) patients with a life expectancy < 1 year, as assessed by a medical oncologist, and poor conditions for radiotherapy or surgery.

The current study patients could be stratified into a late operation group (Group L) if surgery was performed after the 6 months following radiotherapy, and an early operation group (Group E) if surgery was performed within the 6 months following radiotherapy. Several studies reported that the overall median survival time of the MST patients was about 6 months [13,14]. So, we determined early and late operation after radiotherapy based on the period of 6 months from radiotherapy to surgery.

All patients underwent conventional radiotherapy. The clinical target volume was defined as the internal target volume plus 0.3 cm margins for set-up uncertainty. The dose was prescribed for planning the target volume. The biologically effective dose (BED) was calculated to compare different radiotherapy fractionation regions. BED = nD [1 + D/(a/b)], where *n* = fraction number, D = dose per fraction. The most common regimen was 25 Gy in five fractions.

### 2.2. Variables

To analyze the difference between the two groups, we identified demographic factors, tumor grading, operative factors, preoperative radiological findings and clinical factors.

Data on patients demographic factors including age, sex, body mass index, smoking, operation timing from radiotherapy, the cause of the operation (mechanical pain, neurologic deficits), preoperative embolization and comorbidities such as hypertension, diabetes mellitus, liver disease and pulmonary disease were collected and analyzed. Additionally, we analyzed the primary tumor (lung, breast, prostate, kidney, thyroid, hepatic, stomach and colon, sarcoma and others), location (junctional region, mobile, semirigid, rigid), radiosensitivity (radiosensitive, radioresistant), and level of metastasis in spine (one level, two levels, above three levels). Moderately to highly radiosensitive tumors to radiotherapy include most hematologic malignancies (i.e., lymphoma, multiple myeloma and plasmacytoma), as well as selected solid tumors (i.e., breast, prostate, ovarian and neuroendocrine carcinomas and seminoma). However, most solid tumors are radioresistant to radiotherapy, including renal cell carcinoma, colon, non-small cell lung carcinoma, hydroid, hepatocellular carcinoma, melanoma and sarcoma [6]. Tumor grading included Bilsky grade, the spine instability neoplastic score (SINS) and the Karnofsky performance scale [15,16,17]. The Bilsky grade uses T2-weighted magnetic resonance imaging (MRI) to measure spinal cord compression. A Bilsky grade 0 indicates bone-only disease, while grade 1 indicates a range from epidural impingement only to deformation of the thecal sac with spinal cord abutment. Grade 2 indicates spinal cord compression with visible cerebrospinal fluid (CSF) around the cord, whereas grade 3 indicates no visible CSF around the cord. The SINS consists of six parameters including the location of the spinal lesion, the quality of the lesion, spinal alignment, degree of vertebral body collapse, involvement of the posterolateral elements and the presence of the mechanical pain using a simple radiograph, computed tomography and MRI.

Operative factors included operation time, estimated blood loss, transfusion, decompression methods (only posterior decompression, posterior decompression with corpectomy) and fixation level (above and below two levels, above and below three levels). Preoperative radiologic findings included cord compression, pathologic fracture, the extent of vertebral body collapse (above, below 50%), posterolateral involvement, anterior dural compression and tumor type (osteolytic, osteoblastic and mixed lesion) [18]. Cord compression is defined as Bilsky grade 2 or higher. Anterior dura compression is defined as Bilsky grade 1b (thecal sac compression) or higher.

Clinical findings included the preoperative motor grade, postoperative motor grade, preoperative ambulation, postoperative ambulation, hospital stay and final follow-up. Preoperative and postoperative motor grade and ambulation were evaluated at 3 months after the operation, or for patients who did not survive for more than 3 months after the surgery, during the last follow-up. Postoperative complications included symptomatic local recurrence, hematoma, dural tear or neural injury, pleural injury, infection, and wound problems.

### 2.3. Multivariate Logistic Analysis and Subgroup Analysis

To identify the candidates for early operative treatment after radiotherapy, risk factor analysis was performed using a multivariate logistic analysis on possible variables. As subgroup analysis, we analyzed the period from radiotherapy to surgery according to Bilsky grade through survival analysis. Additionally, statistical analysis was performed to identify risk factors for early operation on Bilsky grade 2 patients.

### 2.4. Statistical Analysis

For statistical analysis of univariate parameters, Student’s paired *t*-test, chi-square test, and Fisher’s exact test were used to compare differences in each parameter in univariate analysis. Thereafter, factors with *p* value < 0.1 were considered possible risk factors and used in the multivariate analysis with logistic regression. The backward elimination method in multivariate risk factor analysis was used to identify the risk factors for early operation after radiotherapy. All statistical analyses were performed using SPSS Statistics 21.0 (IBM, Armonk, NY, USA).

## 3. Results

### 3.1. Demographics Factors and Tumor Grading

The average age of the patients was 57.4 ± 10.4 years. Of the total patients, 51 (63.0%) were men, while 30 (37.0%) were women. Patients who underwent surgery within 6 months after radiotherapy classified as the early operation group (group E, *n* = 47, 58.0%), while surgery cases after 6 months after radiotherapy were assigned to the late operation group (group L, *n* = 34, 42.0%). In demographic factors, the operative timing from radiotherapy was significantly earlier in group E than in group L (2.1 ± 3.1 vs. 14.3 ± 7.3; *p* < 0.001). In tumor location, although not statistically significant, the semirigid region was more frequent in group E than group L (26/47, 55.3% vs. 12/34, 35.2%; *p* = 0.075). In contrast, the mobile region was significantly less in group E than group L (6/47, 12.8% vs. 13/34, 38.2%, *p* = 0.008). Among the tumor grading, there were no significant differences between two groups. Detailed contents are described in Table 1.

### 3.2. Operative Factors

The average operation time was 188.1 ± 80.7 min, the mean estimated blood loss was 607.2 ± 532.7 mL, and 25 (30.9%) patients underwent blood transfusion at the perioperative time. Among the operative factors, there were no significant differences between the two groups (Table 2).

### 3.3. Preoperative Radiological Findings

In the preoperative radiologic findings, the ratio of pathologic fracture was significantly higher in group E than in group L (42/47, 89.4% vs. 20/34, 58.8%, *p* = 0.001). In tumor characters, although not statistically significant, the osteolytic lesion was more frequent in group E than group L (37/47, 78.7% vs. 21/34, 61.8%; *p* = 0.095). In contrast, the osteoblastic lesion was less in group E than group L (2/47, 4.3% vs. 5/34, 14.7%, *p* = 0.099). Other details are described in Table 2.

### 3.4. Clinical Findings and Postoperative Findings

The hospital stay was 16.7 ± 10.5 days in group L and 26.3 ± 26.2 days in group E, indicating a longer hospital stay in group E (*p* = 0.048). In addition, the final follow-up was 14.4 ± 12.6 months in group L and 8.7 ± 9.8 in group E, and the follow-up period was shorter in group E (*p* = 0.034). Postoperative complications were not different between the two groups. Other details are described in Table 3.

### 3.5. Multivariate Logistic Analysis and Subgroup Analysis

**Figure 1:**
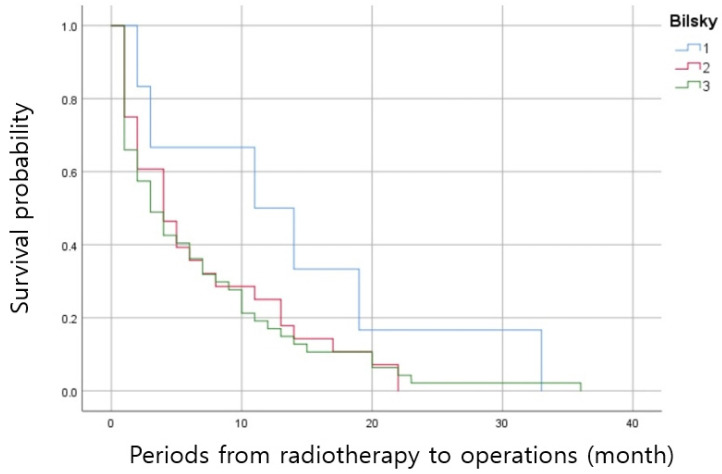
The survival curves about Bilsky grade and the period from radiotherapy to surgery.

**Figure 2:**
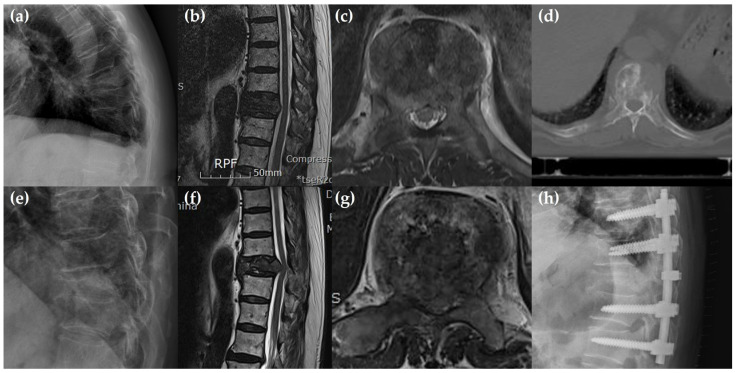
Representative case in group E of an 81-year-old male patient who underwent radiotherapy as the primary treatment for T10 metastasis underlying prostate cancer. (**a**–**d**) Pre-radiotherapy radiograph, CT and MRI revealed the T10 metastasis, Bilsky grade 2 and osteolytic lesion with pathologic fracture. (**e**–**g**) Three months post-radiotherapy, the patient experienced severe back pain and incomplete lower extremities paralysis showing Bilsky grade aggravation (2 -> 3) (**h**) The patient emergently underwent posterior decompression with T8-9-11-12 posterior fusion surgery.

In multivariate logistic regression analysis, pathologic fracture and a semirigid (thoracic) region are the risk factors of early operative treatment after radiotherapy (adjusted odds ratio, 10.524, 4.282; *p* = 0.001, 0.039, Table 4). In subgroup analysis, there was several differences (Bilsky grade 1 vs. 2, 1 vs. 3) in the period from radiotherapy to surgery according to Bilsky grade (Bilsky grade 1, *n* = 6, 13.6 ± 11.4 months; grade 2, *n* = 28, 6.9 ± 6.8 months, grade 3, *n* = 47, 6.6 ± 7.5 months, grade 1 vs. 2; p = 0.049, grade 1 vs. 3; *p* = 0.047, grade 2 vs. 3; *p* = 0.868, Table 5, Figure 1). In the comparative analysis between two groups performed only on Bilsky grade 2 patients, there was a significant difference in the presence of pathologic fracture (Group E vs. Group L; 16/17, 94.1% vs. 6/11, 54.5%, *p* = 0.013, Table 6). A representative case from Group E is illustrated in Figure 2.

## 4. Discussion

Radiotherapy was generally the primary treatment for patients with MST, and nowadays, has become a pillar of multimodality management, allowing for cytoreduction prior to surgery, lesion control following separation surgery and prevention of tumor recurrence following En bloc resection [19]. However, based on the author’s experiences, it was thought that the proportion of patients whose symptoms worsened, or paralysis progressed after radiotherapy, was higher than expected. Therefore, the purpose of this study was to identify patients who would benefit from operative treatment rather than primary radiotherapy. Several studies reported that surgery in addition to radiotherapy over a lifetime was both more effective and less costly than radiotherapy alone, and therefore was found to be cost-effective [11,20]. In addition, Chen B et al. reported that there was no difference in the length of hospital stay between surgery and radiotherapy, but surgery showed better therapeutic efficacy in terms of quality of life, life expectancy and complications [12]. In this way, when radiotherapy fails, it can be disadvantageous in terms of cost-effectiveness and therapeutic efficacy, so we tried to find candidates who would perform better with surgical treatment prior to radiotherapy.

Evidence in the literature supports the argument that radiotherapy is a safe and effective modality for local tumor control, with low associated complication rates in patients with spinal metastasis [7]. In addition, stereotactic radiosurgery, an advanced form of conventional radiotherapy, can be used as a treatment regardless of tumor histology, which has expanded the role of radiotherapy [21]. However, several problems could arise after radiotherapy, and there was typically vertebral compression fracture because of iatrogenic damage caused by the high radiation doses used during index treatment [22,23,24]. Previous studies have shown that vertebral compression fracture could occur after radiotherapy in up to 36% of patients [22]. It has been reported that vertebral compression fracture is dose-dependent on radiotherapy and occurs more at 20 Gy or more [23]. The known risk factors are older age, osteolytic lesion and spinal malalignment. Patients with these risk factors were recommended to receive only 16 to 18 Gy [24]. In our study, the early operation rate after radiotherapy was higher in the presence of compression fracture, which could be understood in the same context as these previous reports.

According to the multivariate logistic regression analysis of this study, semirigid (thoracic) regions were identified as a risk factor for early operative treatment after radiotherapy in MST patients. The spinal canal at the thoracic region is narrower than at other regions, and for this reason, the spinal cord is vulnerable to space-occupying lesions in this region [25]. Additionally, the thoracic region has fewer radiculomedullary arteries and poorer collaterals than other regions, resulting in poor blood supply [26,27]. Therefore, in this region, the spinal cord function could quickly deteriorate in MST patients if there is cord compression. Falavigna et al. reported that in a comparison surgery and radiotherapy between external immobilization and radiotherapy in thoracic MST patients, the ambulation function was maintained, and pain was significantly more reduced in the surgery and radiotherapy group than patients who underwent immobilization and radiotherapy [28]. Park et al. reported that the thoracic level in MST patients was associated with poor postoperative neurological recovery [29].

The surgical treatment of patients with MST aims at pain relief and the improvement of neurological deficit through nerve decompression and mechanical stabilization. Since most patients do not complain of severe symptoms, surgical treatment is not necessarily performed primarily. According to the decision-making system, high-grade epidural spinal cord compression accompanied by neurology is an indication for primary operative treatment [30]. However, according to this study, even if the neurology is mild or the cord compression is not a very high grade (not Bilsky grade 3), early operative treatment after radiotherapy could occur in patients with pathologic fracture or thoracic lesions in Bilsky grade 2 or higher (adjusted odds ratio, 10.524, 4.282; *p* = 0.001, 0.039).

According to one systematic review, in patients with a solid tumor, the cumulative incidence of metastatic spinal cord compression ranges from 0.29 to 10.00% (mean 2.84%, 95% confidence interval [CI]: 1.54–4.14%) [31]. And in patients with spinal metastasis, the cumulative incidence of pathologic compression fracture ranges from 5.30 to 74.5% (mean 25.44%, 95% CI: −26.78–77.66%) [31]. In this study, the rates of cord compression (80.2%) and pathologic fracture (76.5%) were much higher than general cases, because only the patients who underwent surgical treatment were targeted. In these patients, the possibility of early operative treatment after radiotherapy should be closely examined.

The early operation group had a longer hospital stay and a shorter final follow-up than the effective group. Several studies have shown that postoperative complications may be associated with a longer hospital stay after surgery in MST patients, but in this study, there was no statistically significant difference in postoperative complications between the two groups [32,33]. However, Ramos et al. reported that the probability of major complications, including pneumonia, occurring after surgery in MST patients was 19.3%, and among these, the presence of pathologic fracture and admission type (elective vs. emergent) were significantly associated with the occurrence of major complications [34]. There was a significant difference in the presence of pathologic fracture between the two groups, so it could be thought that it was related to the length of hospital stay and final follow-up period.

The Bilsky grade was first used by Bilsky et al. to communicate the degree of spinal cord compression [15]. According to Bendfeldt et al., an increased length of hospital stay, many nonhome discharges, poor surgical outcomes and low 1-year survival rates were reported in patients with a higher Bilsky grade than a low grade [35]. According to the neurologic, oncologic, mechanical and systemic (NOMS) criteria, surgical treatment could be considered for patients with high Bilsky grades, but prioritizing surgical treatment in patients with mild symptoms might be a burden on patients and surgeons. However, the period from radiotherapy to surgery was significantly different between grades 1 and 2, and grades 1 and 3. In particular, in the case of Bilsky grade 2 or higher, surgical treatment was performed at about 6 months, so it could be said that radiotherapy might be ineffective. Therefore, Bilsky grade 2 could be considered an important factor in determining the treatment plan. Additionally, as confirmed in the multivariate analysis, in cases with pathologic fractures or semirigid (thoracic) lesions, it would be better to consider surgical treatment primarily.

### Limitations

The first limitation of this study is that only patients who underwent surgical treatment were targeted, and selection bias could intervene. However, unlike other studies, this study tried to reduce bias by collecting data from four institutions. It is suggested that further research would improve this study if it included a comparative analysis of early operation after radiotherapy in patients who did not undergo surgery. Secondly, due to the relatively small sample size, caution is required when interpreting statistical analysis. Therefore, additional study using a larger sample size as a larger multi-center study is needed. Additionally, while research was conducted on primary tumors, histology was not analyzed in this study, which also warrants further investigation in this subject. Finally, the study analyzed patients who mainly underwent posterior decompression and conventional radiotherapy, but further research would be beneficial if similar studies were conducted on patients who receive different surgical methods such as en bloc resection and separation surgery, as well as stereotactic radiosurgery.

## 5. Conclusions

Although this may be limited to only patients who underwent surgery, early operative treatment after radiotherapy is highly likely in patients with metastatic spinal tumor accompanied by a Bilsky grade above 2, pathologic fracture and semirigid (thoracic) lesions. In these patients, it might be better to consider surgical treatment as the primary treatment.

## Figures and Tables

**Table 1:** Demographic factors and tumor grading between the study groups.

	Group E	Group L	*p* Value
	*n* = 47	*n* = 34	
Demographic factor			
-Age	57.2 ± 11.5	57.6 ± 8.9	0.856
-Sex			0.512
-Male	31 (65.9)	20 (58.8)	
-Female	16 (34.1)	14 (41.2)	
-Body mass index	23.5 ± 6.2	23.8 ± 5.9	0.802
-Smoking	7 (19.3)	7 (13.7)	0.532
-Operative timing from radiotherapy	2.1 ± 3.1	14.3 ± 7.3	<0.001 *
-Cause of the operation			
-Neurological deficits	30 (63.8)	23 (67.6)	0.721
-Mechanical pain	39 (83.0)	29 (85.3)	0.779
-Preoperative embolization	23 (48.9)	14 (41.2)	0.489
-Hypertension	16 (35.5)	15 (41.4)	0.357
-Diabetes mellitus	7 (14.9)	9 (26.5)	0.363
-Liver disease	11 (23.4)	6 (17.6)	0.530
-Pulmonary disease	5 (10.6)	6 (17.6)	0.364
Primary cancer			0.261
-Lung	12 (25.5)	6 (17.6)	
-Breast	4 (8.5)	4 (11.7)	
-Prostate	3 (6.4)	2 (5.9)	
-Kidney	2 (4.2)	8 (23.5)	
-Thyroid	1 (2.1)	0 (0.0)	
-Hepatic	11 (23.4)	9 (26.5)	
-Stomach, colon	5 (10.6)	2 (5.9)	
-Sarcoma	2 (4.2)	0	
-Others	7 (14.9)	3 (8.8)	
Location			0.025 *
-Junctional region (cervico-occipital, cervicothoracic, thoracolumbar)	15 (31.9)	9 (26.8)	0.596
-Mobile (cervical, lumbar)	6 (12.8)	13 (38.2)	0.008 *
-Semirigid (thoracic)	26 (55.3)	12 (35.2)	0.075
-Rigid (sacrum)	0	0	<0.001 *
Radiosensitivity			0.872
-Radiosensitive	9 (19.1)	7 (20.6)	
-Radioresistant	38 (80.9)	27 (79.4)	
Level of metastasis in spine			0.344
-One level	29 (61.7)	26 (76.5)	
-Two levels	6 (12.8)	2 (5.9)	
-Above three levels	12 (25.5)	6 (17.6)	
Tumor grading			
-Bilsky grade 1	2 (4.2)	4 (11.7)	0.203
Grade 2	17 (36.2)	11 (32.4)	0.721
Grade 3	28 (59.6)	19 (55.9)	0.740
-SINS	11.1 ± 3.2	10.0 ± 2.8	0.107
-Karnofsky performance scale	58.7 ± 10.7	58.5 ± 17.7	0.951

SINS, spine instability neoplastic score. * *p* < 0.05.

**Table 2:** Operative factors and preoperative radiological findings between the study groups.

	Group E	Group L	*p* Value
	*n* = 47	*n* = 34	
Operative factors			
-Operation time	184.5 ± 85.3	193.0 ± 74.9	0.635
-Estimated blood loss	480.0 ± 575.5	627.7 ± 629.4	0.338
-Transfusion	20 (42.6)	17 (50.0)	0.429
-Transfusion amount	1307.2 ± 1637.7	972.6 ± 672.6	0.388
-Decompression methods			0.166
-Posterior decompression + corpectomy	13 (27.7)	5 (14.7)	
-Posterior decompression	34 (72.3)	29 (85.3)	
-Fixation levels			0.757
-Above and below two levels	45 (95.7)	33 (97.1)	
-Above and below three levels	2 (4.3)	1 (2.9)	
Preoperative radiological findings			
-Cord compression	45 (89.4)	30 (67.6)	0.203
-Pathologic fracture	42 (89.4)	20 (58.8)	0.001 *
-Vertebral body collapse			0.308
-50% <	28 (59.6)	24 (70.6)	
-50% >	19 (40.4)	10 (29.4)	
-Posterolateral involvement	42 (89.4)	31 (91.2)	0.787
-Anterior dura compression	45 (93.6)	29 (79.4)	0.099
-Tumor characters			
-Osteolytic lesion	37 (78.7)	21 (61.8)	0.095
-Osteoblastic lesion	2 (4.3)	5 (14.7)	0.099
-Mixed lesion	8 (17.0)	8 (23.5)	0.468

* *p* < 0.05.

**Table 3:** Clinical findings and postoperative findings between the study groups.

	Group E	Group L	*p* Value
	*n* = 47	*n* = 34	
Clinical findings			
-Preoperative motor grade	3.9 ± 1.1	3.7 ± 1.2	0.495
-Postoperative motor grade -Preoperative ambulation	4.3 ± 0.9	4.0 ± 1.3	0.234
	31 (66.0)	25 (73.5)	0.467
-Postoperative ambulation	34 (72.3)	26 (76.5)	0.675
-Hospital stay	26.3 ± 26.2	16.7 ± 10.5	0.048 *
-Final follow up	8.7 ± 9.8	14.4 ± 12.6	0.034 *
Postoperative complication			
-Local recurrence	10 (21.3)	9 (26.5)	0.586
-Hematoma	2 (4.3)	1 (2.9)	1.000
-Dural tear, nerve injury	2 (4.3)	0	0.509
-Pleural injury	1 (2.1)	1 (2.9)	1.000
-Infection	2 (4.3)	1 (2.9)	1.000
-Wound problem	2 (4.3)	2 (5.8)	1.000

* *p* < 0.05.

**Table 4:** Multivariate logistic regression analysis of risk factors related to early operative treatment after radiotherapy.

	B	Adjusted Odds Ratio	95% CI	*p* Value
Semirigid lesion	1.103	4.282	1.060–8.570	0.039
Pathologic fracture	2.103	10.524	2.299–29.189	0.001 *
Osteolytic lesion	1.023	3.320	0.926–8.363	0.068

CI: confidence interval, * *p* < 0.05. * After univariate analysis, factors with *p* value < 0.1 were considered possible risk factors and used in the multivariate analysis with logistic regression using the backward elimination method.

**Table 5:** Periods from radiotherapy to surgery according to the Bilsky grade.

	N	Periods from Radiotherapy to Surgery (mo)	*p* Value
-Bilsky grade			
-Bilsky 1	6	13.6 ± 11.4	0.049 *
-Bilsky 2	28	6.9 ± 6.8	
-Bilsky 1	6	13.6 ± 11.4	0.047 *
-Bilsky 3	47	6.6 ± 7.5	
-Bilsky 2	28	6.9 ± 6.8	0.868
-Bilsky 3	47	6.6 ± 7.5	

* *p* < 0.05.

**Table 6:** Risk factor analysis between the study groups in Bilsky grade 2.

	Group E	Group L	*p* Value
	*n* = 17	*n* = 11	
Operation from radiotherapy	2.4 ± 1.5	13.9 ± 5.7	<0.001 *
Operation due to pain	16 (94.1)	10 (90.9)	0.747
Operation due to neurologic deficit	8 (47.1)	8 (72.7)	0.180
Semirigid lesion	12 (70.6)	6 (54.5)	0.387
Pathologic fracture	16 (94.1)	6 (54.5)	0.013 *
Cord compression	17 (100.0)	11 (100.0)	1.000
Vertebral body collapse	4 (23.5)	2 (18.2)	0.736
Anterior dural compression	16 (94.1)	8 (72.7)	0.114
Osteolytic lesion	14 (82.3)	6 (54.5)	0.112
Osteoblastic lesion	1 (5.9)	1 (9.1)	0.747

* *p* < 0.05.

## Data Availability

The data presented in this study is available on request from the corresponding author.
